# Increased OCT3 Expression in Adipose Tissue With Aging: Implications for Catecholamine and Lipid Turnover and Insulin Resistance in Women

**DOI:** 10.1210/endocr/bqad172

**Published:** 2023-11-16

**Authors:** Fozia Ahmed, Milica Vranic, Susanne Hetty, Argyri Mathioudaki, Vagia Patsoukaki, Giovanni Fanni, Maria J Pereira, Jan W Eriksson

**Affiliations:** Department of Medical Sciences, Clinical Diabetology and Metabolism, Uppsala University, 751 85 Uppsala, Sweden; Department of Medical Sciences, Clinical Diabetology and Metabolism, Uppsala University, 751 85 Uppsala, Sweden; Department of Medical Sciences, Clinical Diabetology and Metabolism, Uppsala University, 751 85 Uppsala, Sweden; Department of Medical Sciences, Clinical Diabetology and Metabolism, Uppsala University, 751 85 Uppsala, Sweden; Department of Medical Sciences, Clinical Diabetology and Metabolism, Uppsala University, 751 85 Uppsala, Sweden; Department of Medical Sciences, Clinical Diabetology and Metabolism, Uppsala University, 751 85 Uppsala, Sweden; Department of Medical Sciences, Clinical Diabetology and Metabolism, Uppsala University, 751 85 Uppsala, Sweden; Department of Medical Sciences, Clinical Diabetology and Metabolism, Uppsala University, 751 85 Uppsala, Sweden

**Keywords:** OCT3, adipose tissue, estradiol, aging, T2D

## Abstract

**Background:**

Catecholamine-stimulated lipolysis is reduced with aging, which may promote adiposity and insulin resistance. Organic cation transporter 3 (OCT3), which is inhibited by estradiol (E2), mediates catecholamine transport into adipocytes for degradation, thus decreasing lipolysis. In this study, we investigated the association of *OCT3* mRNA levels in subcutaneous adipose tissue (SAT) with aging and markers of insulin resistance in women.

**Methods:**

SAT biopsies were obtained from 66 women with (19) or without (47) type 2 diabetes (age 22-76 years, 20.0-40.1 kg/m^2^). OCT3 mRNA and protein levels were measured for group comparisons and correlation analysis. SAT was incubated with E2 and *OCT3* mRNA levels were measured. Associations between *OCT3* single nucleotide polymorphisms (SNPs) and diabetes-associated traits were assessed.

**Results:**

OCT3 mRNA and protein levels in SAT increased with aging. SAT from postmenopausal women had higher levels of OCT3 than premenopausal women, and there was a dose-dependent reduction in *OCT3* mRNA levels in SAT treated with E2. *OCT3* mRNA levels were negatively associated with markers of insulin resistance, and *ex vivo* lipolysis. *OCT3* SNPs were associated with BMI, waist to hip ratio, and circulating lipids (eg, triglycerides).

**Conclusion:**

OCT3 mRNA and protein levels in SAT increased with aging, and mRNA levels were negatively associated with markers of insulin resistance. E2 incubation downregulated *OCT3* mRNA levels, which may explain lower *OCT3* mRNA in premenopausal vs postmenopausal women. High OCT3 protein levels in adipose tissue may result in increased catecholamine degradation, and this can contribute to the reduction in lipolysis observed in women with aging.

Aging is often characterized by adipose tissue dysfunction, which is implicated in the development of both obesity and type 2 diabetes (T2D) (1). The sympathetic nervous system in part controls adipose tissue function through the release of catecholamines, such as noradrenaline (2). Noradrenaline plays an important role in adipocyte lipid mobilization, as it regulates the breakdown of triglycerides into free fatty acids and glycerol, a process known as lipolysis, and this occurs through binding beta-adrenergic receptors (2). The sympathetic tone is determined by the release, transport, and degradation of catecholamines (2). There are 2 distinct forms of catecholamine transport: neuronal transport and extraneuronal transport. Neuronal transport involves catecholamine uptake to and from nerve endings and this is mainly through the transporters SLC6A2, SLC6A3, and SLC6A4 (3, 4). Extraneuronal transport is mainly mediated by organic cation transporters (OCTs). *OCT3* (*SLC22A3)* was identified in a genome-wide association study as a novel locus with significantly different effects on body mass index (BMI) in younger vs older individuals (5). Moreover, Paquette et al found that an *OCT3* single nucleotide polymorphism (SNP) was associated with lipoprotein (a) and cardiovascular disease in individuals with familial hypercholesterolemia, highlighting a possible link between OCT3 expression and dyslipidemia and obesity (6).

OCT3 has been implicated in adipocyte lipolysis and browning (3). Gao et al recently showed that there is an age-induced reduction in catecholamine-stimulated lipolysis in subcutaneous adipose tissue (SAT) from women, which may be due to an upregulation of enzymes related to catecholamine degradation (7). A reduction in noradrenaline-stimulated lipolysis impairs lipid turnover and promotes adipose tissue accumulation, through triglyceride accumulation (hypertrophy) which are features observed with aging and obesity (7). Interestingly, estradiol, which declines in women after menopause, has been shown to be an inhibitor of OCT3-mediated transport (8). The role of OCT3 in human SAT lipid and glucose metabolism has yet to be well investigated. In this study, we assess the association between OCT3 mRNA and protein levels in SAT with aging, in addition to assessing the association between *OCT3* mRNA levels in SAT with markers of obesity and insulin resistance. Understanding whether altered OCT3 expression explains changes in adipose tissue lipolysis may provide insight into a potential target for preventing age-related changes in body fat distribution and the development of insulin resistance.

## Methods and Subjects

### Subjects

Abdominal SAT was obtained by needle biopsies from 66 women with (*n* = 19) or without (*n* = 47) T2D, in order to obtain subjects with a wide range of insulin sensitivity and resistance. Needle biopsies were obtained by aspiration from the lower part of the abdomen after local dermal anesthesia with lidocaine (Xylocaine, AstraZeneca, Sodertalje, Sweden). The anthropometric characterization (Table 1) of subjects included measurements taken after an overnight fast, with fasting blood samples collected to assess levels of glycated hemoglobin (HbA1c), glucose, lipids, and insulin. Postmenopausal status was defined as a period of 12 months or longer since the last menstruation. One premenopausal woman was taking oral contraceptives (dienogest/etynilestradiol) and one was on a hormone birth control patch (etynilestradiol/norelgestromin). One postmenopausal woman was treated with systemic hormonal replacement therapy (estriol) and one was treated with local hormonal pessary (estradiol). The subjects with T2D were prescribed a stable dose of metformin for a minimum of 3 months. Additionally, an oral glucose tolerance test was conducted to analyze plasma glucose, insulin, and free fatty acids (FFA). Body composition analysis was performed using whole-body magnetic resonance imaging (MRI), all as previously reported (9). Subjects with type 1 diabetes, other endocrine disorders, cancer, or other major illnesses, as well as ongoing medication with beta-adrenergic blockers, systemic glucocorticoids, or immune-modulating therapies were excluded from the study.

**Table 1.** Anthropometric and clinical characteristics of participating women

	Without T2D	T2D
N	47	19
Age (years)	54 ± 16	52 ± 10
Menopause (Y/N)	33/14	12/7
BMI (kg/m^2^)	27.4 ± 4.4	34.3 ± 5.1
WHR	0.9 ± 0.1	1.0 ± 0.1
Plasma glucose (mmol/L)	5.7 ± 0.9	8.1 ± 1.6
HbA1c (mmol/mol)	32.1 ± 11.2	51.8 ± 11.0
HbA1c (% NGSP)	5.1 ± 1.0	6.9 ± 1.0
Serum insulin (mU/L)	8.8 ± 5.9	22.2 ± 11.2
HOMA-IR	2.3 ± 1.5	7.8 ± 4.4
Total cholesterol (mmol/L)	5.1 ± 1.7	5.0 ± 0.8
Plasma HDL cholesterol (mmol/L)	1.8 ± 0.7	1.1 ± 0.7
Plasma LDL cholesterol (mmol/L)	3.4 ± 1.1	3.2 ± 0.7
Plasma triglycerides (mmol/L)	1.2 ± 0.4	1.9 ± 1.0

Data are mean ± SD. Blood chemistry is fasting.

Abbreviations: BMI, body mass index; HbA1c, glycated hemoglobin; HDL, high-density lipoprotein; HOMA-IR, homeostatic model assessment for insulin resistance; LDL, low-density lipoprotein; NGSP, National Glycohemoglobin Standardization Program; T2D, type 2 diabetes; WHR, waist to hip ratio.

Part of the adipose tissue biopsy was snap-frozen in liquid nitrogen and used for RNA-Seq. In addition, SAT biopsy was used for adipocyte and stromal vascular fraction (SVF) isolation. Isolated mature adipocytes were used for adipocyte size measurements and *ex vivo* analyses of lipolysis and glucose uptake. Not all experiments could be performed in every subject due to the limited amount of aspired adipose tissue. The Regional Ethics Review Board in Uppsala (Dnr 2013/330 and Dnr 2013-183/494) and Gothenburg (Dnr 336-07) approved the studies, and all participants gave their written informed consent.

### Magnetic Resonance Imaging

MRI was used to assess volumes of abdominal SAT, visceral adipose tissue (VAT), and liver fat percentage as previously described (9).

### Adipose Tissue Incubations

Adipose tissue (*n* = 8) was incubated at 37 °C using phenol red–free DMEM media containing 6mM glucose (Invitrogen Corporation, Paisley, OR, USA), 10% charcoal-stripped fetal bovine serum (FBS, Invitrogen), and 1% penicillin-streptomycin (PEST, Invitrogen) in the presence or absence of 1nM to 100nM (physiological to supraphysiological levels) estradiol (E2) (Sigma, Saint Louis, MO, USA) for 24 hours. At the end of the incubation period, tissue was collected and snap-frozen for mRNA analysis. Part of the incubated tissue was used to perform lipolysis in isolated adipocytes.

### Human Preadipocyte Differentiation into Mature Adipocytes

Preadipocytes were isolated from the SVF of the SAT as previously described (9, 10). Human preadipocytes were differentiated for up to 14 days, as previously reported (10, 11). The cells were seeded at 10 000 to 30 000 cells/cm^2^ into 12 well plates (Thermo Fisher) using preadipocyte media. After cells reached 100% confluence, they were cultured for 1 additional day, after which adipogenesis was induced by adding a differentiation cocktail (phenol red–free DMEM/Ham's F12, 1% penicillin-streptomycin, 100nM human insulin (Actrapid, Novo Nordisk), 17µM pantothenate (Sigma), 33µM biotin (Sigma), 1µM cortisol (Sigma), 1µM rosiglitazone (Sigma), 250µM 3-isobutyl-1-methylxanthine (IBMX, Sigma), 10µg/mL transferrin human (Sigma), 2nM 3, 3, 5-triiodo L-thyronine (T3, Sigma)) for 5 days with replacement of induction cocktail on day 3. On day 5, cells were then cultured in maintenance medium (composition is the same as the differentiation cocktail except for omitting IBMX) until day 14. Medium was replenished every 3 days.

### Gene Expression Analysis

Gene expression was assessed using either RNA-Seq or quantitative polymerase chain reaction (qPCR).

#### Transcriptomics

A subgroup of SAT samples was used for RNA-Seq at Exiqon A/S (9), and Novogene (12). RNA-Seq data are presented as Fragment Per Kilobase of transcript per Million mapped reads (FPKM). FPKM is a unit used in RNA-Seq analysis, normalizing gene expression by accounting for both gene length and total number of mapped reads; thus, it is a measure of gene expression that allows for comparisons between different genes and across samples. The data were used to determine correlations between *OCT3 (SLC22A3)* mRNA levels and markers of lipid and glucose metabolism.

#### Quantitative PCR

Total RNA was extracted from whole adipose tissue (*n* = 8) using the RNeasy lipid tissue mini kit (Qiagen). The total RNA concentration and purity were measured with the Nanodrop 2000 spectrophotometer (Thermo Fisher Scientific, Rockford, IL, USA). RNA (400 ng) was then reverse-transcribed using High-Capacity cDNA Reverse Transcription Kit (Applied Biosystems, Thermo Fisher Scientific, CA, USA). The protocols were carried out as per the manufacturers' guidelines. TaqMan gene expression assays (Thermo Fisher) were used to study the expression of *OCT3* (Hs01009571_m1). Gene expression was detected using the QuantStudio 3 sequence detection system (Applied Biosystem) and calculated using a 2^−deltaCT^. The gene expression levels were normalized to the housekeeping gene *GUSB* (Hs00939627_m1), and all samples were run in duplicates.

### Isolation of Human Adipocytes and SVF for Immunoblotting

Adipocytes and SVF were isolated from SAT (*n* = 3) for analysis of OCT3 protein, as described previously (13, 14). Adipose tissue (500 mg) was digested using collagenase A (Roche) in a shaking water bath at 105 rpm for 1 hour at 37 °C in Medium 199 (Gibco, Life Technologies) supplemented with 6mM glucose, 4% bovine serum albumin (BSA, Sigma), 150nM adenosine (Sigma), pH = 7.4. The floating adipocytes were washed 4 times in Hank's medium (6 mmol/L glucose, 4% BSA, 0.15 μmol/L adenosine, pH = 7.4) and were separated from the medium by filtration through dinonyl phthalate (Merck, Darmstadt, Germany). The SVF fraction was isolated from the collagenase medium by centrifugation for 3 minutes at 1200 rpm and washed with phosphate-buffered saline (PBS). The adipocytes and SVF were used for immunoblotting analysis of OCT3. Total protein levels of the adipocyte and SVF were measured using the BCA protein assay kit (Pierce, Thermo Scientific) and used to extrapolate the total OCT3 protein levels in the whole tissue sample.

### Western Blot

Total protein was isolated from adipocytes, SVF, preadipocytes, and differentiated cells on day 14 of *in vitro* differentiation (*n* = 3), as previously reported (11). Cells were lysed in ice-cold lysis buffer: 25mM Tris-HCl (Sigma), pH 7.4; 0.5mM EGTA (Sigma); 25mM NaCl (Sigma); 1% Nonidet P-40; 10mM NaF; 100nM okadaic acid (Alexis Biochemicals), 1×Complete protease inhibitor cocktail (Roche, Indianapolis, IN, USA) and 1mM orthovanadate (Sigma). The lysate was vortexed and incubated on ice for 10 minutes and then centrifuged at 15 000*g* for 15 minutes at 4 °C. The infranatant was transferred into a new tube and saved at −80 °C. Proteins (10-20 µg) were separated by sodium dodecyl sulfate–polyacrylamide gel electrophoresis (SDS-PAGE; 5%-8% gradient stain-free gels, Bio-rad), transferred to nitrocellulose membranes and blocked with 0.05% Tween-PBS (Medicago) with 5% BSA (Sigma). Stain-free blot imaging was used to quantify the total protein for each sample and to normalize OCT3 protein levels as described by (15). Membranes were incubated overnight with the primary antibody anti-OCT3 (1:500, Abcam, ab124826, RRID:AB_10974589). Membranes were then washed with 0.05% Tween-PBS and incubated with horseradish peroxidase conjugated goat anti-rabbit secondary antibody (1:2000, Cell Signaling, 7074S, RRID:AB_2099233). Protein bands and stain-free blots were imaged using enhanced chemiluminescence with a high-resolution field and quantified with ChemiDocTM MP System (Bio-Rad) and quantification with Image Lab Software (software version).

### Immunohistochemistry

The SAT was fixed overnight at room temperature in a paraffin solution, which was replaced with 70% ethanol the following day. Immunocytochemistry staining was performed on SAT sections (*n* = 8). Sections were deparaffinized and rehydrated, and this was followed by heat-induced antigen retrieval. In brief, slides were placed in Tris-EDTA buffer (10mM Tris base, 1mM EDTA, 0.05% Tween) and incubated in a water bath at 95 °C for 15 minutes. Slides were washed 2 × 5 minutes with PBS and 0.025% Triton-X-100 and then blocked in PBS with 5% goat serum and 1% BSA 2 hours at room temperature. Slides were incubated with the primary antibody OCT3 (1:100, Abcam, ab124826, RRID:AB_10974589) overnight in a humidified chamber, followed by the goat anti-rabbit secondary antibody conjugated AlexaFluor 488, (1:250, Thermo, A11008, RRID:AB_143165). SAT sections were imaged using the InCellis Fluorescent microscope (Bertin Instruments) and 3 images were used for analysis from each histological section. Images were quantified using ImageJ Version 1.51 and the average mean fluorescence intensity relative to the total area corrected for background for the triplicate images was used as a measure for OCT3 protein levels.

### Glucose Uptake

Glucose uptake in isolated adipocytes (*n* = 34) was performed as described previously (12, 16). In brief, SAT was digested using collagenase A (Roche) in a shaking water bath at 105 rpm for 1 hour at 37 °C in Medium 199 (Gibco, Life Technologies, Paisley, UK) supplemented with 6mM glucose, 4% bovine serum albumin (BSA, Sigma), 150nM adenosine (Sigma), pH = 7.4. The cell suspension was passed through a 250 μm nylon mesh and collected into a Falcon tube. Adipocytes were isolated from digestion media and washed 3 times at 5-minute intervals in glucose-free Krebs-Ringer bicarbonate medium (KRH) supplemented with 4% BSA, 150nM adenosine, pH = 7.4. Next, adipocytes were incubated at 37 °C in a shaking water bath at 65 rpm for 15 minutes in KRH medium without (basal), or with 25 or 1000 µU/mL of insulin (Actrapid, Novo Nordisk, Bagsværd, Denmark). After 15 minutes, the cells were incubated with D-(U-^14^C) glucose (0.86 µM glucose, 0.26 mCi/mL, Perkin Elmer, Boston, USA) for 45 minutes. The reaction was stopped by transferring the reaction tubes onto ice, and cells were separated from the media by centrifugation using silicon fluid (WR Chemicals, Leuven, Belgium). Radioactivity associated with cells was then measured with a scintillation counter (Radiomatic series 500TR, Perkin Elmer Analytical Instruments). Glucose uptake was expressed as clearance of medium glucose per cell. Cell number was determined following measurements of triglyceride content (Doles extraction) (17) and cell size (Axiovision, München, DE) (18).

### Lipolysis

Lipolysis was measured in isolated adipocytes from SAT (*n* = 31-32) as previously described (19). SAT was digested using collagenase and adipocytes were isolated as described above (glucose uptake section). The adipocyte suspension (lipocrit 3%-5%) was incubated in Hank's medium containing 6 mM glucose, 4% BSA, 0.15 μM adenosine, pH 7.4, with or without 0.5 μM of isoproterenol (beta-adrenergic agonist) in a gently shaking water bath at 37 °C for 2 hours. Glycerol released into the medium was used as lipolytic index and normalized per cell number (nmol/10E^5^ cells/h).

### Single Nucleotide Polymorphism Analysis

Associations of *OCT3* SNPs with diabetes and obesity-related traits were assessed using Open Targets Genetics (https://genetics.opentargets.org/). We used publicly available results from the UK Biobank, Genetic Epidemiology Research on Adult and Aging (GERA), Genetic Investigation of ANthropometric Traits (GIANT), and the BioBank Japan, Million Veteran Program (MV). We identified associations with 8 metabolic traits for *OCT3* and therefore, the *P* values were Bonferroni-corrected with a factor of 8. The significance threshold for the resulting *P* values in SNP analyses was *P* < 5 × 10^−8^.

### Statistics

All data are presented as mean ± standard error of the mean (SEM), unless otherwise stated. Normality of data was first assessed and a comparison between paired groups was made using a paired *t* test, or between 3 or more using one-way ANOVA with repeated measures or mixed model effects. For multiple comparison correction, the false discovery rate (FDR) method Benjamini, Krieger, and Yekutieli was used. Spearman's correlation was used to test for bivariate analysis, and significant variables were included in a multilinear regression model to predict the impact of clinical variables on the *OCT3* gene expression. A *P* value of less than .05 was considered statistically significant. All data were analyzed using GraphPad Prism 9.0.2, IBM SPSS version 23, or Visualizations for transcriptomics data were performed in the R statistical software version 4.3.0.

## Results

### Human SAT Abundantly Expresses Machinery for Extraneuronal Catecholamine Transport and Degradation

**Figure 1.**
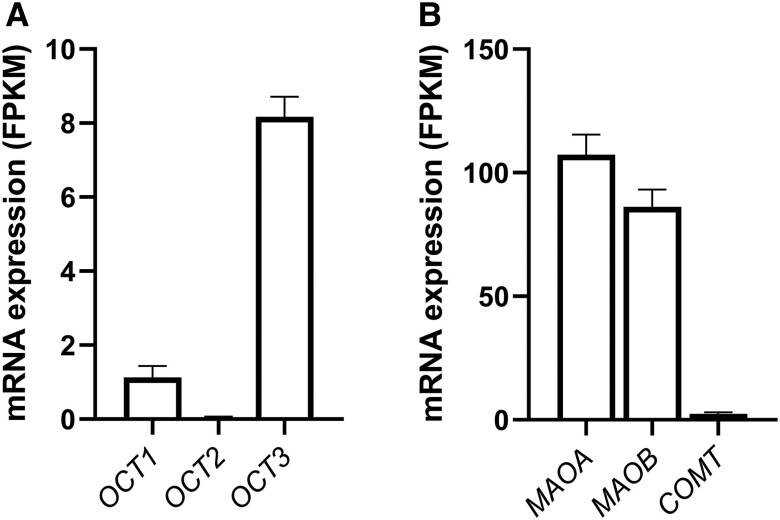
Expression of *OCTs* and catecholamine degradation enzymes in human SAT. (A) Expression of *OCT* transporters in human SAT (*n* = 35). (B) Expression of catecholamine degradation enzymes in SAT (*n* = 26-35). mRNA levels measured in Fragments Per Kilobase Million (FPKM). Data are mean ± SEM.

We assessed the mRNA levels of *OCTs*, which are known extraneuronal catecholamine transporters in SAT and found that *OCT3* mRNA levels were higher than *OCT1* and *OCT2* mRNA levels (Fig. 1A). Furthermore, the mRNA levels of catecholamine degradation enzymes monoamine oxidase A *(MAOA)* and *MAOB* were high compared to catechol-O-methyltransferase *(COMT)* in SAT (Fig. 1B). Due to this, we focused on *OCT3*, (in addition to *MAOA* and *MAOB*) in this study. No significant differences in mRNA levels were found for *OCT1*, *2*, and *3* and *MAOA*, *MAOB*, and *COMT* in subjects with or without T2D (data not shown).

### OCT3 in SAT Is Found Primarily in Mature Adipocytes

**Figure 2.**
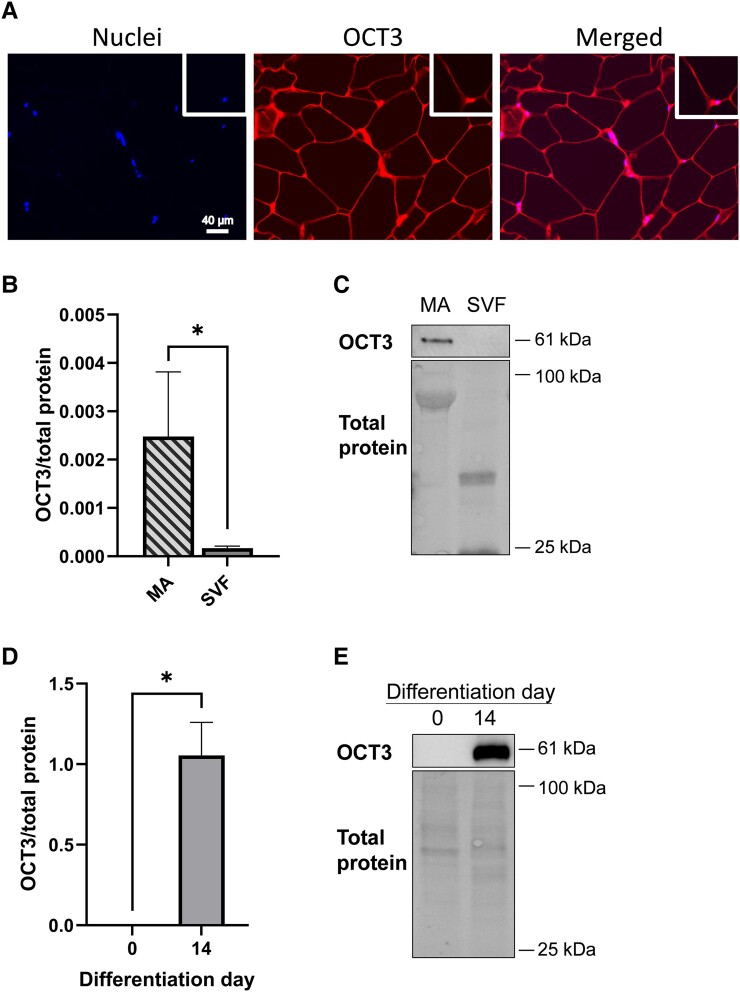
Protein levels of OCT3 in human SAT. (A) Immunofluorescence staining of OCT3 and nuclei in SAT histological samples, 40×magnification (*n* = 1). Scale bar: 40 μm. (B) OCT3 protein quantification and (C) representative immunoblot in MA and SVF paired samples isolated from SAT (*n* = 3). (D) OCT3 protein quantification and (E) representative immunoblot in human subcutaneous primary preadipocytes (day 0) and in *in vitro* differentiated adipocytes (day 14) (*n* = 3). Immunoblot analysis on log-transformed data. Group analysis: *t* test. *P < .05. Data are mean ± SEM. Abbreviations: MA, mature adipocytes; SVF, stromal vascular fraction.

Studies using mice adipocytes have shown that OCT3 was predominantly found in close proximity to the plasma membrane (3), therefore, we assessed whether this was reflected in human SAT. Immunofluorescence imaging of SAT showed that OCT3 was predominately present close to the membrane of mature adipocytes (Fig. 2A). To quantitatively assess the proportion of OCT3 in adipocytes compared to whole adipose tissue, we measured OCT3 protein levels in mature adipocytes and in the SVF fraction of SAT. We found that OCT3 protein levels were approximately 15-fold higher in mature adipocytes than the SVF (Fig. 2B and 2C, *P* < .05). To account for the different proportions of adipocytes and SVF in the SAT sample, we measured total protein and after adjustment, we found that approximately 1% of OCT3 resides in the SVF fraction. Furthermore, we compared OCT3 protein levels in preadipocytes obtained from the SVF at day 0 of differentiation and after 14 days of preadipocyte differentiation, and we found that OCT3 was approximately 10^3^-fold higher in differentiated mature adipocytes (Fig. 2D and 2E, *P* < .05).

### OCT3 mRNA and Protein Levels in SAT Are Increased With Aging and With Menopause

**Figure 3.**
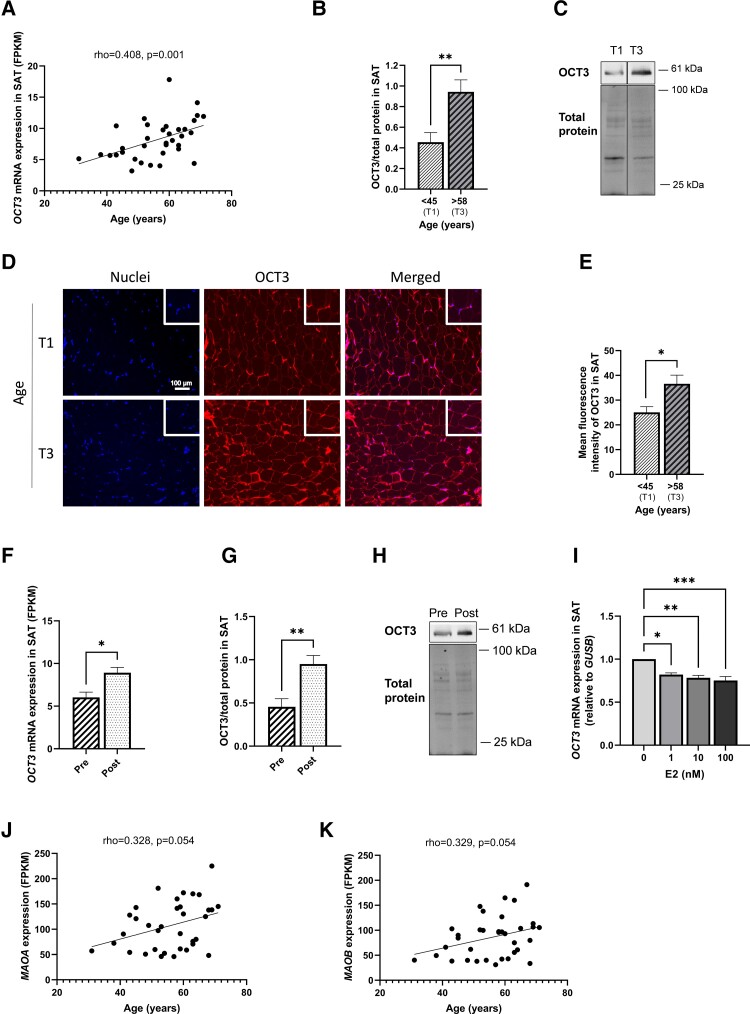
OCT3 mRNA and protein levels are increased with age and menopause in SAT from women. (A) Correlation between *OCT3* mRNA levels in SAT with aging (*n* = 35). (B) Protein quantification and (C) representative immunoblot of OCT3 in SAT from subjects with ages in the lower (T1) and upper (T3) tertile. Bands are derived from 2 parts of same gel (*n* = 12). (D) Fluorescent immunohistochemistry staining and (E) fluorescent quantification of OCT3 and nuclei in SAT histological samples from subjects T1 and T3, 20×magnification (n = 4 for each tertile). Scale bar: 100 μm. (F) mRNA level of *OCT3* in SAT from pre- and postmenopausal women (Pre: *n* = 9; post: *n* = 26). (G) Protein quantification and (H) representative immunoblot of OCT3 in SAT from pre- and postmenopausal women (Pre: *n* = 6; Post: *n* = 10). (I) Effect of 24-hour E2 incubation of SAT on *OCT3* mRNA levels (*n* = 8). Correlation between (J) *MAOA* and (K) *MAOB* mRNA levels in SAT with age (*n* = 35). mRNA levels measured in Fragments Per Kilobase Million (FPKM). Correlation: Spearman's Rho. Group analysis: *t* test. Data are mean ± SEM. *P < .05, **P < .01, ***P < .001. Abbreviations: Pre, premenopausal; Post, postmenopausal.

We assessed the relationship between *OCT3* mRNA levels and aging and found that *OCT3* mRNA levels in SAT were positively correlated with age (Fig. 3A, *P* < .01), and this corresponded to increased OCT3 protein levels in SAT from individuals with ages in the upper tertile compared with the lower tertile (tertile 3 vs 1) (Fig. 3B and 3C, *P* < .01). These differences were also observed with immunofluorescence staining of OCT3 in SAT samples (Fig. 3D and 3E). Correspondingly, OCT3 mRNA and protein levels were higher in SAT from postmenopausal women than from premenopausal women (Fig. 3F, 3G, and 3H, *P* < .05 and *P* < .01, respectively). Estradiol has been reported to be a nonspecific inhibitor of OCT3 (8). Therefore, to assess whether estradiol could account for changes in *OCT3* mRNA levels with aging and menopause, SAT from postmenopausal women was incubated with E2 for 24 hours. The E2 treatment resulted in a dose-dependent reduction in *OCT3* mRNA levels (Fig. 3I, *P* < .05). We also assessed whether mRNA levels of monoamine degradation enzymes were altered with aging and found that *MAOA* and *MAOB* mRNA levels displayed a positive trend with age (Fig. 3J and 3K). Aldo-keto reductases or aldehyde dehydrogenases, which convert primary amines, such as catecholamines, to aldehydes, ammonia, and hydrogen peroxide were unchanged with aging in SAT from women (data not shown).

### *OCT3* mRNA Levels in SAT Are Negatively Associated With Markers of Insulin Resistance

The association between *OCT3* mRNA levels and markers of insulin resistance, dyslipidemia, and adiposity was assessed. We found that *OCT3* mRNA levels in SAT were negatively correlated with area under the curve (AUC) glycerol during an oral glucose tolerance test and markers of insulin resistance (eg, HbA1c, homeostatic model assessment for insulin resistance [HOMA-IR]), and positively associated with high-density lipoprotein (HDL) levels (Table 2, *P* < .05). Levels of *OCT3* mRNA did not significantly correlate to markers of obesity (eg, BMI, waist to hip ratio [WHR]) (Table 2).

**Table 2.** Association between *OCT3* mRNA levels, circulating lipids, and markers of insulin resistance, and adiposity

	Rho	*P* value
*Circulating lipids*		
AUC FFA	−.278	.144
AUC glycerol	**−**.**405**	.036*
Triglycerides	−.266	.122
HDL	.**492**	.003*
LDL	.057	.746
*Insulin resistance*		
HbA1c	**−**.**366**	.048*
HOMA-IR	**−**.**502**	.002*
Plasma glucose	**−**.**369**	.029*
Serum insulin	**−**.**472**	.004*
Matsuda index	.433	.063
*Adiposity*		
BMI	−.287	.095
WHR	−.083	.635
VAT volume^a^	−.407	.094
SAT volume^a^	−.263	.291
VAT/SAT volume^a^	−.123	.627
Liver fat percentage^a^	−.265	.287
Adipocyte size	−.370	.063

Correlation: Spearman's Rho. Significant correlations are shown in bold (*P* < .05). Abbreviations: AUC FFA, area under the curve free fatty acids; area under the curve glycerol; BMI, body mass index; HbA1c, glycated hemoglobin; HDL, high-density lipoprotein; HOMA-IR, homeostatic model assessment for insulin resistance; LDL, low-density lipoprotein; SAT, subcutaneous adipose tissue; VAT, visceral adipose tissue; WHR, waist to hip ratio.

^*^ *P* < .05 (*n* = 29-35).

^*a*^ *n* = 18.

Since age and menopausal status are collinear, we used 2 multiple regression models to assess which characteristics are stronger predictors of *OCT3* mRNA levels in SAT. Model A consists of age, HbA1c, HOMA-IR, and T2D, while model B consists of menopausal status, HbA1c, HOMA-IR, and T2D. The inclusion of either age or menopausal status showed that age appeared to be a stronger predictor of *OCT3* mRNA levels (β = .476 vs β = .305) (Table 3). Moreover, both age and HOMA-IR were independent predictors of *OCT3* mRNA levels (Table 3).

**Table 3.** Regression models for predicting *OCT3* mRNA levels in SAT

	Model A *R*^2^: 0.391		Model B *R*^2^: 0.279	
	Standard β	*P*	Standard β	*P*
Age	.**476**	.005*		
HbA1c	−.083	.717	.068	.789
HOMA-IR	**−**.**463**	.038*	−.470	.053
T2D	.338	.120	.162	.461
Menopausal status			.305	.096

Abbreviations: HbA1c, glycated hemoglobin; HOMA-IR, homeostatic model assessment for insulin resistance; T2D, type 2 diabetes.

^*^ *P* < .05 (N = 35).

### *OCT3* mRNA Levels in SAT Are Negatively Associated With Adipocyte Lipolysis

**Figure 4.**
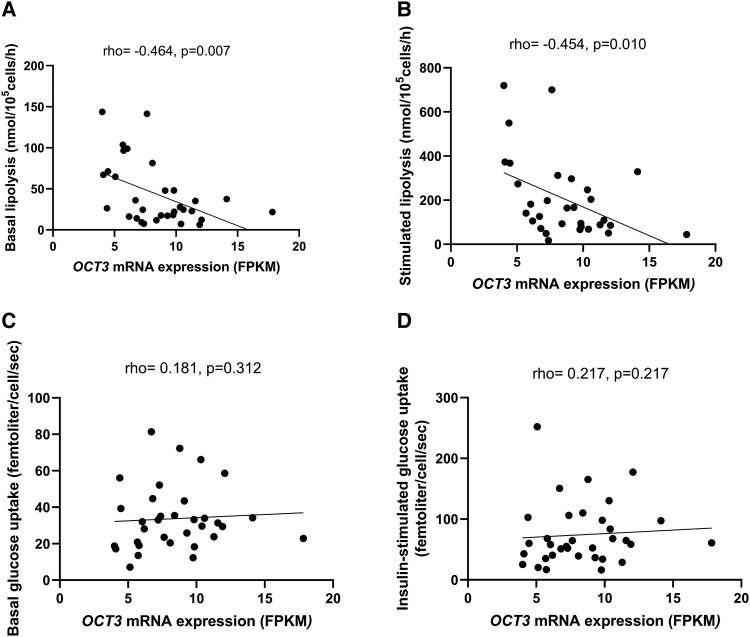
Association between *OCT3* mRNA levels in SAT and *ex vivo* lipolysis and glucose uptake. Association between *OCT3* mRNA levels and (A) basal and (B) isoprenaline-stimulated lipolysis (*n* = 31-32). Correlation between *OCT3* mRNA levels and (C) basal (D) insulin-stimulated (1000 μU/mL) glucose uptake (*n* = 33-34). mRNA levels measured in Fragments Per Kilobase Million (FPKM). Correlation: Spearman's Rho.

Next, we assessed the relationship between *OCT3* mRNA levels and *ex vivo* adipocyte lipolysis and glucose uptake. *OCT3* mRNA levels were negatively associated with basal and isoprenaline-stimulated lipolysis (Fig. 4A and 4B, *P* < .05). This corresponded to a negative association between *OCT3* mRNA levels in SAT with gene markers of lipolysis activation (*ATGL*) and a positive association with lipolysis inhibition (*PLIN1*) (Table 4). Although *OCT3* mRNA levels did not correlate to *ex vivo* adipocyte glucose uptake (Fig. 4C and 4D, *P* > .05), *OCT3* was positively associated with gene markers of insulin signaling and glucose transport markers (*GLUT4* and *AKT1*, Table 4). In addition, *OCT3* mRNA levels were positively correlated with markers of catecholamine degradation (Table 4, *P* < .001).

**Table 4.** Association between *OCT3* mRNA levels and gene markers of lipolysis, glucose uptake, and catecholamine degradation

	Rho	*P* value
*Lipolysis*		
*ADRB2*	.127	.554
*ADRB3*	.224	.196
*ADRA2A*	−.046	.792
*LIPE*	−.279	.104
*ATGL*	**−**.**401**	.047*
*PKA^a^*	−.096	.686
*PLIN1*	.**714**	.001*
*Insulin signaling*		
*GLUT4*	.**348**	.041*
*GLUT1*	.029	.871
*IRS1*	.**674**	.001*
*AKT1*	.**436**	.009*
*AKT2*	.311	.069
*Catecholamine degradation*		
*MAOA*	**.705**	**<**.001*
*MAOB*	**.808**	**<**.001*

Abbreviations: *ADRB2*, beta-2 adrenergic receptor; *ADRB3*, beta-3 adrenergic receptor; *ADRA2A*, alpha-2A adrenergic receptor; *AKT1,* AKT serine/threonine kinase 1; *AKT2,* AKT serine/threonine kinase 2; *ATGL,* Adipose triglyceride lipase; *GLUT4,* glucose transporter 4; *GLUT1,* glucose transporter 1; *IRS1,* insulin receptor substrate 1; *LIPE*, hormone sensitive lipase; *MAOA*, monoamine oxidase A; *MAOB*, monoamine oxidase B; *PKA,* protein kinase A; *PLIN1,* perilipin 1. Correlation: Spearman's Rho.

^*^ *P* < .05 (*n* = 35).

^*a*^ *n* = 20.

### Association of *OCT3* Polymorphisms With Adiposity, Diabetes, and Dyslipidemia

Association between *OCT3* SNPs and obesity and diabetes-related traits were assessed (Table 5). We found that SNPs in *OCT3* were associated with BMI, body fat distribution, WHR, T2D, low-density lipoprotein (LDL), HDL, and triglycerides (Table 5).

**Table 5.** Association of *OCT3* polymorphisms with adiposity, diabetes, and dyslipidemia

Trait	SNP	Effect allele	Other allele	Location	Beta	95% CI	*P* value	Adjusted *P* value	*N*
BMI	rs487152	C	A	Chr. 6:160353454	.017	.012 to .021	2.00E-12	1.60E-11	158 284
	rs518295	G	T	Chr. 6:160354546	.012	.009 to .015	3.00E-09	2.40E-08	523 818
WHR	rs668871	C	T	Chr. 6:160348779	**−**.013	**−**.017 to .001	7.00E-12	5.60E-11	697 734
WHR adjusted for BMI	rs474513	A	G	Chr. 6:160349280	**−**.012	**−**.015 to **−**.008	7.00E-11	5.60E-10	381 152
	rs555754	G	A	Chr. 6:160348391	**−**.023	**−**.023 to **−**.026	1.80E-30	1.44E-29	458 349
	rs501470	T	G	Chr. 6 160349886	**−**.017	**−**.021 to **−**.0140	2.00E-24	1.60E-23	379 501
	rs622217	T	C	Chr. 6:160345738	**−**.019	**−**.023 to **−**.015	4.00E-24	3.20E-23	660 648
	rs673736	G	A	Chr. 6:160355621	**−**.026	**−**.031 to **−**.021	2.00E-20	1.60E-19	219 872
Weight	rs668871	C	T	Chr. 6:160348779	.018	.015 to .021	2.00E-26	1.60E-25	525 535
	rs520829	T	G	Chr. 6:160346873	.261	.180 to .328	4.00E-15	3.20E-14	354 838
Type 2 diabetes	rs501470	T	G	Chr. 6:160349886	**−**.032	**−**.039 to **−**.025	2.00E-17	1.60E-16	1 407 282
	rs543159	C	A	Chr. 6:160354985	**−**.032	**−**.041 to .023	1.00E-12	8.00E-12	1 114 458
LDL cholesterol	rs118039278	G	A	Chr. 6:160564494	.082	.073 to .090	2.00E-79	1.60E-78	416 487
	rs10455872	A	G	Chr. 6:160589086	.088	.077 to .099	3.00E-54	2.30E-53	297 626
	rs1564348	T	C	Chr. 6:160157828	.048	.038 to .058	2.76E-21	2.21E-22	94 595
	rs3125055	T	A	Chr. 6:160315755	.047	.036 to .058	5.92E-16	4.73E-16	94 595
	rs3798221	G	T	Chr. 6:160577116	**−**.046	**−**.058 to **−**.033	1.13E-12	9.00E-12	95 454
	rs12208357	C	T	Chr. 6:160122116	.057	.049 to .065	3.00E-44	2.40E-43	440 546
	rs117733303	A	G	Chr. 6:160501838	.084	.069 to .099	3.00E-27	2.40E-26	440 546
HDL cholesterol	rs571848809	A	T	Chr. 6:160584356	**−**.0557	**−**.063 to **−**.048	3.00E-51	2.40E-50	390 103
	rs11751347	C	T	Chr. 6:160671406	**−**.064	**−**.07 to **−**.053	2.00E-32	1.60E-31	297 626
	rs9457931	A	G	Chr. 6:160508872	**−**.055	**−**.07 to **−**.040	7.30E-13	5.80E-12	94 595
	rs77009508	A	G	Chr. 6:160583940	**−**.063	**−**.070 to **−**.056	3.30E-68	2.60E-67	400 754
	rs9457998	A	G	Chr. 6:160688021	.026	.021 to .032	7.40E-26	5.92E-25	400 754
	rs41267805	G	A	Chr. 6:160500937	**−**.053	**−**.06 to **−**.042	1.10E-23	8.80E-23	400 754
Triglyceride	rs10455872	A	G	Chr. 6:160589086	**−**.052	**−**.051 to **−**.044	2.70E-46	2.16E-45	437 532
	rs9457998	A	G	Chr. 6: 160688021	**−**.037	**−**.042 to **−**.032	2.20E-44	1.76E2-43	437 532
	rs3798220	T	C	Chr. 6: 160540105	**−**.091	**−**.105 to **−**.076	4.40E-37	3.50E.36	437 532
	rs12208357	C	T	Chr. 6: 160122116	.046	.039 to .054	2.60E-34	2.08E-33	437 532
	rs77009508	A	G	Chr. 6: 160583940	.045	.037 to .052	4.00E-32	3.20E-31	441 016
	rs3127599	C	T	Chr. 6: 160486102	.019	.015 to .023	3.70E-21	2.96E-20	437 532
	rs9364554	C	T	Chr. 6: 160412632	.018	.014 to .022	2.80E-18	2.24E-17	437 532

Associations of *OCT3* SNPs with diabetes and obesity-related traits were assessed using Open Targets Genetics.

Abbreviations: BMI, body mass index; HDL, high-density lipoprotein; LDL, low-density lipoprotein; SNP, single nucleotide polymorphism.

### Proposed Outcome Related to Increased OCT3 mRNA and Protein Levels With Aging in SAT From Women

**Figure 5.**
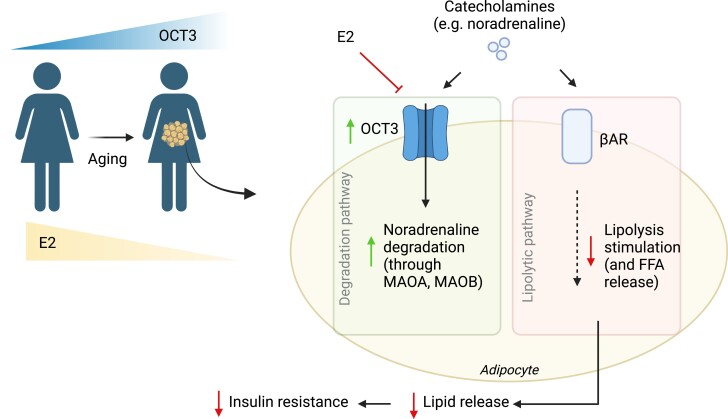
Proposed model illustrating outcome of increased OCT3 mRNA and protein levels in SAT with aging. Our results show that OCT3 mRNA and protein levels are increased in SAT with aging, which may in part be due to reduced E2, which is an inhibitor of OCT3. Increased catecholamine transport and degradation through MAOA and MAOB results in less available noradrenaline to stimulate adipocyte lipolysis, thus reducing the release of lipids from adipocytes. Reduced lipolysis and release of lipids from adipocytes may result in reduced peripheral insulin resistance, highlighting a possible protective role of OCT3. Image created with Biorender.com.

Lastly, we use our data to propose a model of increased OCT3 mRNA and protein levels in SAT with aging (Fig. 5). Our results show that OCT3 is increased in SAT with aging, which may be due to reduced E2, which is an inhibitor of OCT3 (8). Increased catecholamine transport, through OCT3, and degradation, through MAOA and MAOB, would result in less available noradrenaline to stimulate adipocyte lipolysis, thus reducing the release of lipids from adipocytes. Reduced adipocyte lipolysis and release of lipids from adipocytes, in turn, may contribute to reduced peripheral insulin resistance.

## Discussion

Aging is associated with a decrease in catecholamine-stimulated lipolysis of adipocytes, which is linked to an increase in adipose tissue accumulation, hypertrophy, and insulin resistance (7, 20, 21). In this study, we investigated the association between levels of OCT3 in SAT and markers of lipid and glucose metabolism in SAT. To our knowledge, this is the first study to show that in human SAT, OCT3 mRNA and protein levels are upregulated with aging, and *OCT3* mRNA levels are negatively associated with markers of insulin resistance and *ex vivo* adipose tissue lipolysis. Moreover, we found that SAT specimens from postmenopausal women have higher mRNA and protein levels of OCT3 than SAT from premenopausal women, and SAT incubated with E2 for 24 hours has reduced *OCT3* mRNA levels. Our results may indicate that increased OCT3 expression, and subsequent catecholamine degradation, results in reduced catecholamine-stimulated lipolysis observed with aging.

Recent studies have proposed that adipocytes are an important site of noradrenaline clearance in adipose tissue (3, 7). In mice, OCT3 is abundant in white adipose tissue and adipocytes display the greatest rate of noradrenaline clearance (3). Similarly, we found that OCT3 was the predominant extraneuronal catecholamine transporter gene expressed in human SAT, and its gene expression levels were comparable between subjects with and without T2D. Moreover, OCT3 was primarily found close to the membrane of mature adipocytes, which is in agreement with OCT3 localization in mouse primary inguinal adipocytes (3). Histologically, we localize OCT3 in close proximity of the cell surface of adipocytes, but the resolution of our assays does not enable confirmation of OCT3 being exclusively located at the adipocyte plasma membrane rather than in the intercellular space or in adjacent SVF cells. Furthermore, we found abundant mRNA levels of intracellular enzymes for catecholamine degradation, *MAOA*, and *MAOB*, which further indicates that SAT is likely an important site for catecholamine degradation. Our data on the mRNA levels of OCTs and catecholamine degradation enzymes are in accordance with protein levels found in the Human Protein Atlas (22) and previous studies (23‐26).

Since aging is associated with a decrease in catecholamine-stimulated lipolysis, we assessed whether OCT3 mRNA and protein levels were altered with aging in SAT. OCT3 mRNA and protein levels in SAT were increased with aging, and correspondingly, in postmenopausal women. Since age and menopausal status are collinear, we performed 2 multiple regression analysis to determine if the differences we found with menopausal status were mainly due to age. We found that age was a stronger predictor than menopausal status of *OCT3* mRNA levels in SAT. This is in agreement with previous studies showing that markers related to catecholamine degradation were shown to be upregulated in adipose tissue with age (7, 27).

*OCT3* mRNA levels were inversely associated with markers of insulin resistance (ie, HOMA-IR, HbA1c), and our multilinear regression model demonstrated that HOMA-IR was an independent predictor of *OCT3* mRNA levels. This corresponded to a positive association between *OCT3* mRNA levels and gene markers of glucose transport and insulin sensitivity. We hypothesized that high *OCT3* mRNA levels in SAT correspond to reduced capacity to stimulate adipocyte lipolysis, due to increased transport and degradation of catecholamines. We found that *OCT3* mRNA levels were negatively associated with both basal and stimulated lipolysis, and negatively associated with certain gene markers of lipolysis (eg, *ATGL*), emphasizing a possible link between increased *OCT3* mRNA levels and reduced lipolysis. This agrees with a study by Song et al, which showed that adipose-specific OCT3 knockout mice have reduced catecholamine-stimulated lipolysis (3). The relationship between catecholamine-stimulated lipolysis and insulin resistance has been previously shown, and catecholamines are also known to directly impair insulin action by increasing cAMP levels, for example in adipose tissue and skeletal muscle (28‐30). For instance, Mullins et al showed that catecholamine-stimulated lipolysis inhibits adipocyte glucose uptake through inhibition of rapamycin (mTOR) complexes (30). This highlights that increased catecholamine degradation (and thus less stimulation of lipolysis) may be associated to reduced insulin resistance, as we observe in our study.

In mice, aging has been shown to be associated with an upregulation of catecholamine degradation enzymes, *MAOA* and *MAOB*, which correspond to a decrease in serum FFA (27). Since we found that *OCT3* mRNA levels were negatively associated with adipocyte lipolysis, we assessed whether its expression was associated with circulating lipids, and we found that *OCT3* gene expression was negatively associated with AUC glycerol, highlighting a possible link between *OCT3* mRNA levels and circulating lipids. Several genome-wide studies have identified variants in *OCT3* to be associated with lipid traits and cardiovascular risk (6, 31, 32). We assessed whether polymorphisms in *OCT3* were associated with markers of adiposity and insulin resistance and found that *OCT3* polymorphisms were associated with adiposity, diabetes status, and circulating lipids, strengthening the link between OCT3 transport and fat accumulation and insulin resistance.

We found that OCT3 mRNA and protein levels in SAT were positively associated with aging and higher in postmenopausal women. It has been shown that estradiol is a potent inhibitor of OCT3-mediated transport (8). Thus, we assessed whether estradiol treatment of SAT altered *OCT3* mRNA levels. We found that estradiol treatment of SAT resulted in a dose-dependent reduction in *OCT3* mRNA levels. Sex hormones have been shown to be important for the regulation of catecholamine catabolism. For instance, estrogen has been shown to downregulate the expression of the monoamine degradation enzyme *MAOA* in HeLa cells, through estrogen receptor signaling (33). Similarly, in mice, estradiol has been shown to downregulate other monoamine transporters such as PMAT, and correspondingly, serotonin reuptake, through estrogen receptors (34). Estradiol levels are reduced after menopause, which also corresponds to a reduction in adipose tissue lipolysis (7). It is possible that a reduction in estradiol after menopause contributes to an increase in OCT3 mRNA and protein levels in SAT and results in increased catecholamine degradation. To our knowledge, there are no studies investigating the levels of OCT3 in women taking hormone replacement therapy. There have been few studies investigating the direct role of estradiol or estrogen replacement therapy on adipocyte lipolysis, and these studies are often contradictory due to the use of different experimental models. For instance, in one study using ovariectomized mice (a model of menopause), estradiol treatment increased catecholamine-stimulated lipolysis (35), whereas, in a separate study local perfusion of the abdomen with estradiol blunted adipocyte lipolysis (36). Interestingly, in one study performed in humans, hormone replacement therapy (estradiol) increased catecholamine excretion in urine, which was hypothesized to be due to decreased synaptic uptake and decreased catecholamine degradation (37). It is unclear whether the increase in catecholamine excretion and a reduction in degradation may be due to an estrogen-induced reduction of peripheral OCT3 expression and thus decreased catecholamine degradation. Moreover, although it is known that aging and menopause are associated with an increased risk of insulin resistance and the development of T2D (38, 39), it is still not known whether an increase in *OCT3* mRNA levels, and thus a decrease in stimulated lipolysis, could function as a compensatory mechanism to limit insulin resistance. Future studies are needed in order to functionally assess whether estrogen deficiency results in reduced stimulated lipolysis in SAT, such as estrogen treatment in OCT3 knockout ovariectomized mice.

This study has some limitations. This study is explorative in nature and future studies should address the functional role of OCT3 in human adipocytes. However, known inhibitors of OCT3 are nonspecific (40) and target multiple pathways, and are therefore not ideal to assess the role of OCT3 in SAT. Although the relationship between OCT3 and lipolysis has been studied in mice (3) functional studies are warranted in human mature adipocytes to specifically inhibit or knockout OCT3 in order to confirm its importance in lipolysis in the context of aging. Moreover, studies are required to further investigate the role of estrogen on OCT3-mediated catecholamine clearance and lipolysis.

In conclusion, *OCT3* mRNA and protein levels are increased with aging in SAT from women. Moreover, *OCT3* mRNA levels are negatively associated with insulin resistance and *ex vivo* lipolysis. Estradiol treatment of SAT downregulates *OCT3* mRNA levels, which may in part explain lower OCT3 mRNA and protein levels in premenopausal compared with postmenopausal women. This suggests that high OCT3 levels, resulting in increased catecholamine degradation, may partly account for the reduction in adipose tissue lipolysis observed with aging.

## Data Availability

Some or all datasets generated during and/or analyzed during the current study are not publicly available but are available from the corresponding author on reasonable request.

## Abbreviations

AUC: area under the curve
BMI: body mass index
BSA: bovine serum albumin
E2: estradiol
FFA: free fatty acids
FPKM: fragment per kilobase of transcript per million mapped reads
HbA1c: glycated hemoglobin
HDL: high-density lipoprotein
HOMA-IR: homeostatic model assessment for insulin resistance
LDL: low-density lipoprotein
MAO: monoamine oxidase
MRI: magnetic resonance imaging
OCT3: organic cation transporter 3
OGTT: oral glucose tolerance test
PBS: phosphate-buffered saline
RNA-seq: RNA sequencing
SAT: subcutaneous adipose tissue
SEM: standard error of the mean
SNP: single nucleotide polymorphism
SVF: stromal vascular fraction
T2D: type 2 diabetes
VAT: visceral adipose tissue
WHR: waist to hip ratio
